# Polymeric Nanocapsules for Vaccine Delivery: Influence of the Polymeric Shell on the Interaction With the Immune System

**DOI:** 10.3389/fimmu.2018.00791

**Published:** 2018-04-19

**Authors:** Mercedes Peleteiro, Elena Presas, Jose Vicente González-Aramundiz, Beatriz Sánchez-Correa, Rosana Simón-Vázquez, Noemi Csaba, María J. Alonso, África González-Fernández

**Affiliations:** ^1^Inmunología, Centro de Investigaciones Biomédicas (CINBIO) (Centro Singular de Investigación de Galicia), Instituto de Investigación Sanitaria Galicia Sur, Universidade de Vigo, Vigo, Spain; ^2^Department of Pharmacy and Pharmaceutical Technology, School of Pharmacy, University of Santiago de Compostela, Santiago de Compostela, Spain; ^3^Center for Research in Molecular Medicine and Chronic Diseases (CIMUS), University of Santiago de Compostela, Santiago de Compostela, Spain; ^4^Departamento de Farmacia, Facultad de Química, Pontificia Universidad Católica de Chile, Santiago, Chile; ^5^Immunology Unit, University of Extremadura, Cáceres, Spain

**Keywords:** nanocapsules, vaccination, antigen, adjuvant, hepatitis B, rHBsAg, nanovaccines

## Abstract

The use of biomaterials and nanosystems in antigen delivery has played a major role in the development of novel vaccine formulations in the last few decades. In an effort to gain a deeper understanding of the interactions between these systems and immunocompetent cells, we describe here a systematic *in vitro* and *in vivo* study on three types of polymeric nanocapsules (NCs). These carriers, which contained protamine (PR), polyarginine (PARG), or chitosan (CS) in the external shell, and their corresponding nanoemulsion were prepared, and their main physicochemical properties were characterized. The particles had a mean particle size in the range 250–450 nm and a positive zeta potential (~30–40 mV). The interaction of the nanosystems with different components of the immune system were investigated by measuring cellular uptake, reactive oxygen species production, activation of the complement cascade, cytokine secretion profile, and MAP kinases/nuclear factor κB activation. The results of these *in vitro* cell experiments showed that the NC formulations that included the arginine-rich polymers (PR and PARG) showed a superior ability to trigger different immune processes. Considering this finding, protamine and polyarginine nanocapsules (PR and PARG NCs) were selected to assess the association of the recombinant hepatitis B surface antigen (rHBsAg) as a model antigen to evaluate their ability to produce a protective immune response in mice. In this case, the results showed that PR NCs elicited higher IgG levels than PARG NCs and that this IgG response was a combination of anti-rHBsAg IgG1/IgG2a. This work highlights the potential of PR NCs for antigen delivery as an alternative to other positively charged nanocarriers.

## Introduction

Vaccination is one of the most cost-effective health interventions for the prophylaxis of infectious diseases. Conventional vaccines use inactivated or attenuated pathogens—or even subunits of these organisms, such as toxoids and carbohydrates—as antigens (1). Unfortunately, the immune protection provided by these subunit vaccines, even with classical adjuvants, i.e., alum, is often insufficient. Moreover, these vaccines suffer from additional problems, such as limited storage stability and the need for multiple-dose injection schemes to achieve effective protection. Thus, vaccine development has been required to move toward a new generation of vaccines that could provide better and more sustainable protective immune responses (2).

Interest in the use of innovative antigens such as recombinant proteins, peptides, or nucleic acids has increased in the last few decades (3–5). In spite of the many advantages of these new vaccine prototypes, including an enhanced immunogenicity/risk ratio profile and easier manufacture, the development of improved adjuvants that may enhance the potency of these vaccines has become essential. Several inmunostimulant molecules (i.e., cytokines, toll-like receptor ligands, toxins, or saponins) and antigen delivery systems (i.e., virosomes, emulsions, or nanoparticles) are among the potential adjuvants studied in this scope. In particular, the use of nanosystems has attracted significant attention, especially for vaccines requiring an improvement in cell-mediated immune responses (6).

Polymer- and lipid-based nanoparticles have been the most widely explored nanosystems in vaccination. It has already been reported that nanostructures are able to enhance both B and T cell responses due to their similarity with viruses in terms of size and surface properties (7). In addition, due to their prolonged presentation to the immune system, these nanostructures can induce long-lasting immune responses, thereby offering the possibility of decreasing the number of doses required to achieve protective antibody levels. Moreover, nanoparticle formulations have shown the ability to avoid overactivation of the immune response and, simultaneously, to trigger the production of pro-inflammatory cytokines (8).

Our group has developed a number of antigen delivery technologies and validated them using rHBsAg as a model antigen. Namely, we have produced various chitosan-based nanocarriers, including chitosan-coated PLGA nanoparticles (9), CS nanoparticles (10), and CS nanocapsules (NCs) (11). More recently, we also developed arginine-rich based (PARG and PR) NCs (12) and nanoparticles including different anionic polymers (dextran sulfate and alginate) (13). Overall, the *in vivo* administration of these nanocarriers through nasal or parenteral routes has led to significant IgG responses in mice (11, 14). Interestingly, the differences obtained in the elicited immune responses highlighted the importance of the composition of the nanosystem, especially concerning the type of biopolymer exposed on the nanoparticle surface.

The aim of the work described here was to gain an understanding of the role of the polymeric shell of NCs in their interaction with immune cells. For this purpose, three types of NCs with different coating polymers (PARG, PR, and CS), and the corresponding nanoemulsion (NE), were compared in a systematic study. This included the *in vitro* evaluation of cellular uptake, the reactive oxygen species (ROS) production, the activation of the complement cascade, the cytokine secretion profile, the MAP kinases/nuclear factor κB (NFκB) activation, and gene expression. The influence of size, composition of the outer shell layer, and superficial charge in these processes were analyzed, and the two most promising prototypes were selected to perform *in vivo* studies with the rHBsAg antigen. In addition to the systematic study, the four different prototypes were converted into freeze-dried products, to enhance the stability of the formulations under storage and avoid the need of the maintenance of the cold chain. The physicochemical characterization of the resulting products is disclosed in Supplementary Material.

## Materials and Methods

### Materials

Three polymers were used: protamine from Yuki Gosei Kogyo, Ltd. Company (Japan), chitosan hydrochloride salt (Protasan UP CL113 deacetylation degree of 75–90%, *M*_w_: 125 kDa) was purchased from NovaMatrix (Sandvika, Norway) and polyarginine (PARG) (*M*_w_ 5–15 kDa) was obtained from Sigma-Aldrich (Barcelona, Spain). Miglyol^®^ 812, a mixture of three different triglycerides of medium chain fatty acids, was obtained from Sasol GmbH (Witten, Germany). Recombinant hepatitis B surface antigen (rHBsAg) (*M*_w_ 24 kDa) in an aqueous suspension [0.16 mg/mL in phosphate-buffered saline (PBS)] was kindly donated by Shantha Biotechnics Limited (Hyderabad, India). PEG-stearate (Simulsol^®^ M52) was purchased from Invitrogen (UK). Cobra venom factor (CVF) was supplied by Quidel Corporation (USA), and the PVDF membranes were obtained from Bio-Rad (USA).

Aluminum hydroxide, sodium hydroxide, rhodamine 6G, Triton 100x, PBS, 5-bromo-4-chloro-3-indolyl phosphate (BCIP), glycerol, sodium cholate, 2ʹ,7-dichlorofluorescein diacetate (DCFH-DA), lipopolysaccharide (LPS), phorbol myristate acetate (PMA), and phytohemagglutinin (PHA) were all obtained from Sigma-Aldrich (USA).

Cell culture media (DMEM and RPMI 1640) and the antibiotics penicillin and streptomycin were supplied by Gibco (Life Technologies, Scotland). Heat inactivated fetal bovine serum (FBS) and Accutase^®^ were purchased from PAA Laboratories (Austria), and Ficoll-Hypaque was obtained from GE Healthcare (Sweden).

#### Antibodies

##### Enzyme-Linked Immunosorbent Assay (ELISA) for Recombinant Hepatitis B Antigen (rHBsAg)

Enzyme-linked immunosorbent assay kit (Murex HBsAg Version 3) was obtained from Murex Biotech Ltd. (Dartford, UK), and the polyclonal chicken antibodies against HBsAg were purchased from Abcam (UK). Mouse and rabbit antibodies against HBsAg used in the ELISA test were purchased from Acris Antibodies GmbH (Hiddenhausen, Germany) and Biokit (Barcelona, Spain), respectively. Secondary mouse antibodies against IgG, IgG1, IgG2a conjugated with horseradish peroxidase (HRP) were acquired from Southern Biotech (USA).

##### Western Blot and ELISA for Complement Factors (C3 and C5)

Polyclonal secondary antibodies used in the Western blot assay were purchased from Dako (Glostrup, Denmark), and the monoclonal antibody (mAb) against complement factor C3 was obtained from Abcam (UK). Human C5a ELISA Kit II BD OptEIA™ used for the detection of complement factor C5 was purchased from BD Biosciences Pharmingen (CA, USA).

##### Flow Cytometry: Cell Markers and Cytokines

Monoclonal antibodies for flow cytometry assays were anti-CD3-PECy5.5, anti-CD19-PECy5.5 (Southern Biotech, AL, USA), anti-HLA-DR-FITC (Beckman Coulter, USA), and anti-CD71-PE (NIT Zipper, Nanoimmunotech, Spain).

Cytokine detection was performed with the FlowCytomix kit Th1/Th2 from eBioscience (San Diego, CA, USA).

##### Analysis of Kinase Routes by Western Blot

Rabbit monoclonal antihuman p-extracellular signal-regulated kinase (ERK), p-p38, p-SAP/JNK, IKBα, and goat anti-rabbit IgG-HRP antibodies used for the Western blot analysis were supplied by Cell Signaling Tech. (Danvers, MA, USA), and the anti-GAPDH antibody was obtained from Sigma-Aldrich (Barcelona, Spain).

### Nanoparticle Preparation

Nanocapsules were prepared using the solvent displacement technique previously described by our research group (15). Briefly, a mixture of 12 mg of PEG-st and 5 mg of sodium cholate was dissolved in 0.750 mL of ethanol, followed by the addition of 62.5 µL of Miglyol^®^ 812 to the previous mixture. The volume of the organic phase was increased by adding 4.25 mL of acetone. This organic phase was added to an aqueous phase composed of 10 mL of a solution of 0.05% w/v of PR, PARG, or CS for the preparation of the PR and CS NCs or 10 mL of milliQ water in the case of the control, uncoated NE. In all cases, the nanostructures were spontaneously formed upon diffusion of the solvents. The nanostructures were isolated by ultracentrifugation of the samples at 61,690 *g* for 1 h at 15°C (Optoma TM L-90K Ultracentrifuge, Beckman Coulter, USA).

### Physicochemical Characterization of the Nanostructures

The hydrodynamic diameter and polydispersity index (PDI) of the NCs and the corresponding NE were measured by photon correlation spectroscopy. Zeta potential was determined by laser-Doppler anemometry (Zetasizer^®^, NanoZS, Malvern Instruments, Malvern, UK). Samples were measured after diluting in milliQ water (970 µL of water: 30 μL of NCs) or in 1 mM KCl for size and zeta potential, respectively.

### Fluorescent Labeling of the Nanocarriers

Nanocapsules were prepared as described in Section “Nanoparticle Preparation” by adding an aliquot of the chromophores to the oily phase. Rhodamine B, rhodamine 6G, and DiD (1,1′-dioctadecyl-3,3,3′,3′-tetramethylindodicarbocyanine, 4-chlorobenzenesulfonate salt) were selected as fluorescent markers at two different concentrations (10 and 50 µg/mL dissolved in ethanol), evaluating both the influence of the nature of the chromophore and its concentration. The evaluation of the encapsulation efficiency of the chromophores was carried out indirectly by quantifying the non-encapsulated fluorophore remaining in the undernatants after the centrifugation step. Rhodamines were measured at an emission wavelength of 590 nm (LB 940 Multimode Reader Mithras, Berthold Technologies GmbH & Co KG, Germany); UV–VIS spectroscopy at λ = 646 nm (Du-BoLife Science UV/VIS Beckman Coulter) was used to quantify DiD. The three different fluorophores were incorporated into the NE, PR and CS NCs, assuming no differences in the release and the encapsulation efficiency between the PR and the PARG NCs. The evaluation of the loading efficiency and release profile of the different fluorescent dyes are disclosed in Supplementary Material (Supplementary Figure 1 and Supplementary Table 1).

### Association of Hepatitis B Surface Antigen (rHBsAg) With the NCs

PR and PARG NCs, prepared as described in Section “Nanoparticle preparation,” were incubated with rHBsAg in equal volumes at a mass ratio of 4:1 (theoretical concentration of the cationic polymer adsorbed on the NC surface:rHBsAg) to achieve adsorption onto the polymeric corona. The process was performed under mild conditions (RT, 1 h), and the loaded NCs were subsequently isolated (30 min, 12,872 *g*, 15°C). The amount of rHBsAg associated to the NCs was indirectly quantified by measuring the concentration of antigen remaining in the undernatant after the ultracentrifugation step. An ELISA commercial kit was used to quantify the rHBsAg concentration in the samples, according to the manufacturer’s instructions. The association efficiency for rHBsAg (A.E.%) was calculated indirectly as the difference between the concentration of free antigen detected in the supernatant and the total concentration in the initial suspension.

### Freeze Drying of the NCs

To obtain a lyophilized product, preliminary studies were carried out to determine the best conditions to preserve the original physicochemical properties of the prototypes upon reconstitution (Supplementary Figure 2). The conversion into a dry powder was performed using a freeze-drying process (Genesis SQ Freeze dryer, Virtis, USA) (11). The studies were conducted with aliquots of the four nanosystems in a range of concentrations (0.25–1% w/v) both with and without cryoprotectant (glucose, trehalose, and sucrose at 5 and 10% w/v). Based on the results obtained, and to standardize the process conditions for the four prototypes, a solution of each of the different nanosystems (0.75% w/v) was frozen overnight at −20°C in the presence of sucrose as cryoprotectant (10% w/v). Subsequently, the samples were transferred to the lyophilizer (Genesis SQ Freeze Drier, Virtis, USA) and submitted to an initial drying step for 24 h at −35°C and 2–10 mTorr followed by a second and a third drying step (24 h, 0°C and 16 h, 20°C, respectively). After the process, the freeze-dried cake had an overall good aspect and could be resuspended swiftly in ultrapure water.

### Activation of the Complement Cascade

The activation of the complement cascade upon contact with the different nanostructures was analyzed by two methods: degradation of the complement C3 factor by Western blot and quantification of the C5a levels by ELISA. A pool of human plasma from healthy donors was incubated with different concentrations of nanosystems (10, 100, and 1,000 µg/mL of the constituent polymer and the corresponding amount of NE) and veronal buffer (pH 7.4). An aliquot of 50 µL of each formulation and controls were incubated at 37°C for 1 h. CVF and PBS were used as positive and negative controls, respectively. After the incubation step, samples were centrifuged at 16,000 *g* for 30 min to separate the nanosystems from the other components.

#### Qualitative Determination by Western Blot

After the centrifugation step, supernatants containing the complement proteins were loaded onto a 10% SDS-PAGE gel and transferred to a PVDF membrane with the Transblot-Turbo Transfer System (Bio-Rad, Hercules, CA, USA). Membranes were blocked overnight at 4°C with TBST with 5% of non-fat dried milk. Membranes were then incubated with a mouse mAb against human C3 (1:1,000) (90 min, RT). A secondary incubation step was performed under the same conditions (90 min, RT) with secondary polyclonal goat anti-mouse IgG antibodies conjugated with alkaline phosphatase (1:2,000). Intensive washes were carried out between all steps. Membranes were revealed with BCIP. To quantify the degradation, the intensity of the lower band was normalized to the negative control, where some basal C3 degradation was observed.

#### Quantitative Determination by ELISA

An ELISA assay was performed using the Human C5a ELISA Kit II to confirm the activation of the complement cascade quantifying C5a factor levels. Briefly, standards and samples were added for 2 h at RT to the wells previously coated with an mAb against the C5a factor. After several washes, wells were incubated for 1 h with a mixture of biotinylated antihuman C5a antibody and streptavidin-HRP. Intensive washes were performed between all these steps. To detect the presence of the antibody-antigen complexes, TMB Substrate Reagent was added. After 30 min of incubation, the reaction was stopped, and the absorbance was measured at 450 nm.

### *In Vitro* Cell Studies

#### Cells

Raw 264.7 (mouse macrophage cell line), Jurkat (human leukemic T-cell line), Hmy (human B lymphoblast cell line), and HL60 (human promyelocytic leukemia cell line) were purchased from ATCC (American Type Culture Collection, Middlesex, UK). All lines were cultured in RPMI supplemented with 10% (v/v) of heated-inactivated FBS, 2 mM glutamine, and 100 U/mL of penicillin/streptomycin, at 37°C in a 5% CO_2_ atmosphere. Cells were split every other day to maintain 70–80% confluent cultures.

Human peripheral blood mononuclear cells (hPBMCs) were obtained from three healthy voluntary donors. To separate mononuclear cells, 15 mL of EDTA-anticoagulated blood was diluted with 15 mL of PBS and centrifuged (180 *g*, 30 min, 20°C) through a Ficoll-Hypaque gradient using a 7:3 ratio (diluted blood:ficoll). Mononuclear cells were collected at the interface between the ficoll and the plasma and washed twice by centrifugation (100 *g*, 5 min, 20°C) with complete medium.

#### Cell Viability Assay: xCELLigence^®^ System

An xCELLigence^®^ RTCA DP Instrument (Roche Diagnostics, Penzberg, Germany) was used according to the manufacturer’s instructions to analyze cell viability. Raw 264.7 cells were cultured at a density of 1.5 × 10^4^ cells/well with 200 µL of RPMI supplemented with 10% FBS until they reached the exponential phase (37°C and 5% CO_2_) (around 18 h). The different prototypes were added in a range of six different concentrations from 250 to 7.8 µg/mL. As negative controls, cell culture media and the blank nanosystems were added to the wells. The impedance was monitored at 15 min intervals for 72 h.

#### Cellular Uptake by Macrophages

To evaluate the internalization of the nanosystems by fluorescence microscopy, 1 × 10^5^ of Raw 264.7 cells were seeded in an NUNC 96-Well Optical-Bottom Plates with Coverglass Base (Thermo Fisher Scientific, Langenselbold, Germany) with RPMI 10% FBS in the presence and absence of the fluorescent prototypes at 10 and 50 µg/mL of the constituent polymer and the corresponding amount of NE for 30 min. Three washes were performed to remove the excess nanosystems, and cells were observed with an inverted fluorescence microscope (IX50, Olympus Optical Co. GmbH, Hamburg, Germany).

A similar protocol was followed to perform the flow cytometry analysis. In this case, after incubation with the fluorescently labeled prototypes followed by three washes with PBS, cells were detached using Accutase^®^ (10 min, 37°C and 5% CO_2_). Finally, cells were washed once with complete medium to inactivate Accutase^®^, and the suspension was analyzed by flow cytometry (Accuri Cytometers, Ann Arbor, MI, USA).

A kinetic and more detailed study of the internalization was carried out by Confocal Laser Scanning Microscopy (Leica SP5) using the High Content Screening Automation HCS A module and the LAS AF MATRIX software. Images were acquired every 5 min during 3 h.

#### Cytokine Profile Evaluation

Cytokine production was assessed by incubating 2 × 10^5^ hPBMCs during 24 h in 96-well plates (37°C, 5% CO_2_) in the presence of the prototypes at two different concentrations: 10 and 100 µg/mL. As negative and positive controls, cells were incubated with complete medium or with a combination of a solution of 1 µg/mL of LPS and 10 µg/mL of PHA, respectively. After 24 h, the plate was centrifuged (100 *g*, 5 min, 4°C), and supernatants were collected and stored at −20°C before analysis. The cytokine levels (IL-12p70, INFγ, IL-2, IL-10, Il-8, IL-6, IL-4, IL-5, IL-1β, TNFα, and TNF β) were quantified using the FlowCytomix kit Th1/Th2 according to the manufacturer’s instructions. Briefly, 25 µL of antibody-coated microspheres was incubated with 25 µL of culture supernatants and 50 µL of biotin-conjugated secondary antibodies (2 h, RT) using a microplate shaker. After several washes, 50 µL of streptavidin conjugated to phycoerythrin and 100 µL of PBS-T were added to the preparation and incubated on a microplate shaker (1 h, RT). Finally, phycoerythrin-bound beads were studied by flow cytometry (FC500, Beckman Coulter, Miami, FL, USA), and data were analyzed using FlowCytomix Pro 3.0 Software (eBioscience, San Diego, CA, USA).

#### ROS Production

The production of intracellular ROS was detected by measuring the oxidation of 2′,7-dichlorofluorescein diacetate (DCFH-DA). This marker can be oxidized by ROS and converted to a fluorescent compound.

The human promyeloblast cell line HL60 was used to evaluate the production of ROS. 2.5 × 10^5^ cells were cultured in 24-well plates in contact with 10 or 100 µg/mL of the different prototypes after 1 and 12 h. As negative and positive controls, complete medium and a solution of 10 µM of PMA were used, respectively. After this incubation step, cells were collected and centrifuged (100 *g*, 5 min) and resuspended in PBS containing 5 µM of DCFH-DA. Afterward, the samples were incubated at 37°C during 30 min. Finally, cells were washed twice with PBS and analyzed by flow cytometry (Accuri Cytometers, Ann Arbor, MI, USA). The median of the fluorescence intensity was normalized to the negative control.

#### Activation Markers

Changes in the pattern of expression of membrane markers were studied in hPBMCs (CD3, CD19, CD71, and HLA-DR). 2 × 10^5^ cells were seeded in a 96-well plate and incubated with and without the nanosystems at two different concentrations (10 and 100 µg/mL of the constituent polymer and the corresponding amount of the NE) during 24 h. As a positive control, a solution of 10 µg/mL of PHA was used. Cells were labeled with the antibodies during 30 min at 4°C. In a final, the cells were washed twice with PBS and analyzed by flow cytometry (FC500, Beckman Coulter, Miami, FL, USA).

#### Routes of Activation. MAP Kinases and NFκB

The study of the signaling pathway activation (ERK1/2, p38, SAPK/JNK and NFκB) was performed with the human tumoral cell lines Jurkat and Hmy. These two cell lines were incubated with 10 µg/mL of the constituent polymer of PR NCs and PARG NCs for 1 and 3 h.

To perform the Western blot analysis, cells were washed with PBS and then suspended in a lysis buffer (Tris–HCl 10 mM, pH 8, NaCl 150 mM, EDTA 2.5 mM and 1% NP-40) containing a protease and phosphatase inhibitor (Complete Mini and PhosphoStop from Roche Ltd., Basel, Switzerland). Cell lysates were centrifuged (16,000 *g*, 4°C, 5 min) using an Eppendorf 5415R centrifuge (Eppendorf AG, Hamburg, Germany) to remove cell residues.

Cell extracts were loaded onto a 10% SDS-PAGE gel and transferred to a PVDF membrane using the Transblot-Turbo Transfer System (Bio-Rad, Hercules, CA, USA). PVDF membranes were blocked with 5% of non-fat dried milk in TBST (1 h, RT). Membranes were washed and incubated overnight at 4°C with specific rabbit monoclonal antihuman p-ERK, p-, and p-SAP/JNK antibodies to determine the phosphorylation of ERK, p38, and SAP/JNK kinases (dilutions from 1:10,000 to 1:20,000 in TBST) and with anti IKBα (1:10,000), which is the NFκB inhibitor, to evaluate its activation indirectly. After intensive washes, membranes were incubated with the anti-GAPDH antibody as internal control (1:40,000) for 1 h at RT. Goat anti-rabbit IgG antibodies conjugated to HRP diluted 1:50,000 in TBST with 2.5% of skimmed milk were used as secondary antibodies.

Membranes were revealed with the Clarity Western ECL Substrate (Bio-Rad Laboratories Inc.), and the protein bands were analyzed and quantified using the ChemiDoc XRS imaging system (Bio-Rad Laboratories Inc.).

### *In Vivo* Studies

#### Animals

Female BALB/c mice (4–5 weeks old) were housed in filter-top cages with a 12 h light/12 h dark cycle with a constant temperature environment of 22°C. Food and water were provided *ad libitum*.

Groups of five animals were randomly distributed and immunized twice (0 and 28 days) with 60 µL of PR NCs or PARG NCs incorporating 10 µg of rHBsAg. The injection was performed intramuscularly (i.m.) in the hind leg of the mouse and rHBsAg adsorbed to aluminum hydroxide was used as a positive control. Aluminum hydroxide and rHBsAg solutions were incubated in a volumetric ratio of 3:1 (rHBsAg:alum) for 30 min at 4°C under moderate agitation. The suspension was centrifuged (10,000 *g*, 10 min, 4°C), and the pellet was resuspended in a suitable volume of isotonic saline solution. Blood samples were collected from the mouse maxillary vein at 6 and 10 weeks for the quantification of HBsAg-specific antibodies.

#### Quantification of rHBsAg-Specific IgG and IgG Subtypes by ELISA

96-Well plates were coated with rHBsAg diluted in carbonate buffer (pH 9.6) at a concentration of 5 µg/mL and incubated overnight at 4°C. The plates were then blockaded with PBS-BSA 1% for 1 h at 37°C to reduce the non-specific interactions. A mouse monoclonal IgG anti-HBsAg was used to quantify the levels of specific rHBsAg IgG: the μg/mL of the specific IgG were converted into international units using a solution of anti-HBsAg rabbit IgG with a known concentration into mIU/mL. The controls and a pool of serum samples from each group were serially diluted and incubated for 2 h at 37°C. Two secondary antibodies (goat anti-mouse and anti-rabbit IgG conjugated with HRP) were added and incubated with the samples for 1 h at 37°C. Bound antibodies were revealed with 2,2′-azino-bis(3-ethylbenzothiazoline-6-sulfonic acid) (ABTS), and the optical density was read at 405 nm.

To calculate the ratio IgG1/IgG2a, a pool of sera collected from the immunized mice were analyzed using the same ELISA protocol, but using the goat anti-mouse IgG1 and IgG2a (conjugated to HRP) as secondary antibodies.

#### Restimulation of Splenocytes *Ex Vivo* for Quantitative PCR (qPCR) Gene Expression Assays

Changes in gene expression were evaluated by qPCR using TaqMan^®^ Gene Expression Assays for several genes and the TaqMan^®^ Fast Advanced Master Mix from Life Technologies™. The qPCR was performed in a 7900HT Fast Real-Time PCR System (AB, Life Technology Co.).

Mice previously immunized with two intramuscular doses (0, 4 weeks) were sacrificed at the end of the study (11 weeks after the first immunization). To obtain cell suspensions, spleens were removed under sterile conditions, and cells were dissociated by gentle teasing in complete medium (DMEM with 10% FBS) and filtered with a cell strainer (Falcon, NJ, USA). The suspension was centrifuged (100 *g*, 5 min), and the pellet was resuspended in 7 mL of DMEM. The same protocol followed to obtain hPBMCs was carried out to eliminate spleen erythrocytes and granulocytes.

Mononuclear cells separated by gradient centrifugation at a concentration of 5 × 10^6^ cells/mL were restimulated with 10 µg/mL of rHBsAg for 12 or 24 h. RNA was extracted and purified using the ReliaPrep™ RNA Miniprep Systems kit (Promega), and genomic DNA was eliminated with the same kit. The cDNA was synthetized using the Maxima First Strand cDNA Synthesis kit (Thermo Fisher Scientific Inc.). To check the optimal concentration of cDNA per sample, a qPCR for GAPDH was previously performed at different dilutions.

Data from three mice per group were normalized to the internal control GAPDH, and ΔΔCt was calculated using as a baseline the control data from mice immunized with PBS 1× and restimulated *in vitro* with rHBsAg. These data were averaged, and relative quantification (RQ) was calculated.

### Statistical Analysis

Results are presented as Mean ± SD. Statistical comparisons were made by the Mann–Whitney *U* test for *in vivo* experiments and *T* test for *in vitro* experiments. The accepted level of significance was a *p* value < 0.05.

### Ethical Issues

Institutional ethics approval to work with human samples from healthy donors was obtained from the Ethics Committee for Clinical Research (Xunta de Galicia, Spain, 2013/272). All participants included in the study gave their written informed consent.

All protocols developed with mice were adapted from the guidelines of the Spanish regulations (Royal Decree 53/2013) regarding the use of animals in scientific research and under the approval of the Ethical Committee of the University of Vigo.

## Results and Discussion

### Development and Characterization of Polymeric NCs

The method selected for the preparation of the nanostructures was the solvent displacement technique previously described by our research group (16). The procedure is based on the nanoprecipitation of the lipid components upon the diffusion of a polar solvent into an aqueous phase, where the cationic polymer interacts with the oily nanodroplet cores, thus forming a polymeric coating that surrounds the oily core. Polycationic NCs made of CS (11, 14), PARG (17) and PR (12) with different oil/surfactant ratios were previously developed by our research group. In this work, the type and the ratio of the lipidic constituents were adjusted to develop nanocarriers with the identical oily core composition but varying the polymeric surface coatings. As a result, three different NC formulations and the corresponding negatively charged NE were developed (Table 1). As expected, the three NC formulations had a positive zeta potential and their size varied depending on the nature of the polymer shell. PR and PARG NCs presented zeta potential values close to +30 mV, whereas the value for CS NCs was higher (+47 mV), probably due to the presence of a larger number of positive groups on their surface. With respect to the particle size, PR or PARG NCs had similar sizes, within the range 250–300 nm, and a low PDI (0.2 and 0.1, respectively). By contrast, CS NCs had a larger size and higher PDI (456 ± 2; 0.3). This result can be explained by the different entanglement of the tensioactive agents (PEG-stearate and sodium cholate) with the cationic polymers at the interface of the oily cores. In fact, CS NCs with reduced sizes could also be produced by adjusting the surfactant composition (11).

**Table 1:** Physicochemical characterization of the selected nanocapsules (NCs).

Formulation	Abbreviation	Size (nm)	PDI	Zeta potential (mV)	rHBAg association (%)
Nanoemulsion	NE	264 ± 10	0.2	–23 ± 2	–
Chitosan nanocapsules	CS NCs	456 ± 2	0.3	+47 ± 2	–
Protamine nanocapsules	PR NCs	274 ± 4	0.2	+27 ± 4	–
rHBsAg-loaded protamine nanocapsules	PR NCs-rHBsAg	358 ± 20	0.3	+5 ± 4	74 ± 5
Polyarginine nanocapsules	PARG NCs	251 ± 2	0.1	+30 ± 2	–
rHBsAg-loaded polyarginine nanocapsules	PARG NCs-rHBsAg	320 ± 8	0.3	+15 ± 1	70 ± 7

*PDI, polydispersity index; HBsAg, hepatitis B surface antigen*.

### Loading of rHBsAg Antigen Onto the NCs

Based on the results obtained in the different cellular studies, PR and PARG NCs were selected for loading with rHBsAg. The incorporation of the antigen into the nanostructure surface was attributed to electrostatic forces occurring between the cationic polypeptide of the polymer coating and the negatively charged antigen (−20 mV), as well as to hydrophobic interactions between the antigen and the tensioactive molecules. To associate rHBsAg to the NC surface, the antigen solution was incubated with the previously isolated NCs (11). The mass ratio selected was 4:1 (theoretical concentration of the cationic polymer adsorbed into the NC surface:rHBsAg). Quantification of the non-associated antigen was performed by ELISA. Both prototypes showed an association efficiency greater than 70% (Table 1). Regarding the physicochemical properties of the nanostructures, the association of the antigen to the NC surface led to a size increase and a decrease in the zeta potential. These changes support the presence of the antigen on the NC surface, a location that would theoretically facilitate recognition by antigen-presenting cells (18, 19).

### Activation of the Complement Cascade

The complement system is one of the most important constituents of the innate humoral system. It includes different serum proteins that are activated in cascade upon contact with different stimuli. Three different routes (classic, alternative, and lectins) can activate this system, and the degradation of the C3 is a common step in all of them. The stimulation and the consequent activation of the cascade can promote phagocytosis processes, it can mediate inflammation, and it is involved in the recognition and clearance of pathogens (20). The complement serves as a first line of defense, and it plays an important role in promoting antigen-specific responses by enhancing both B- and T-cell immunity (21). In fact, the bioactivity of alum (the most used adjuvant in vaccines worldwide) is strongly related to its ability to activate the complement cascade (22).

**Figure 1:**
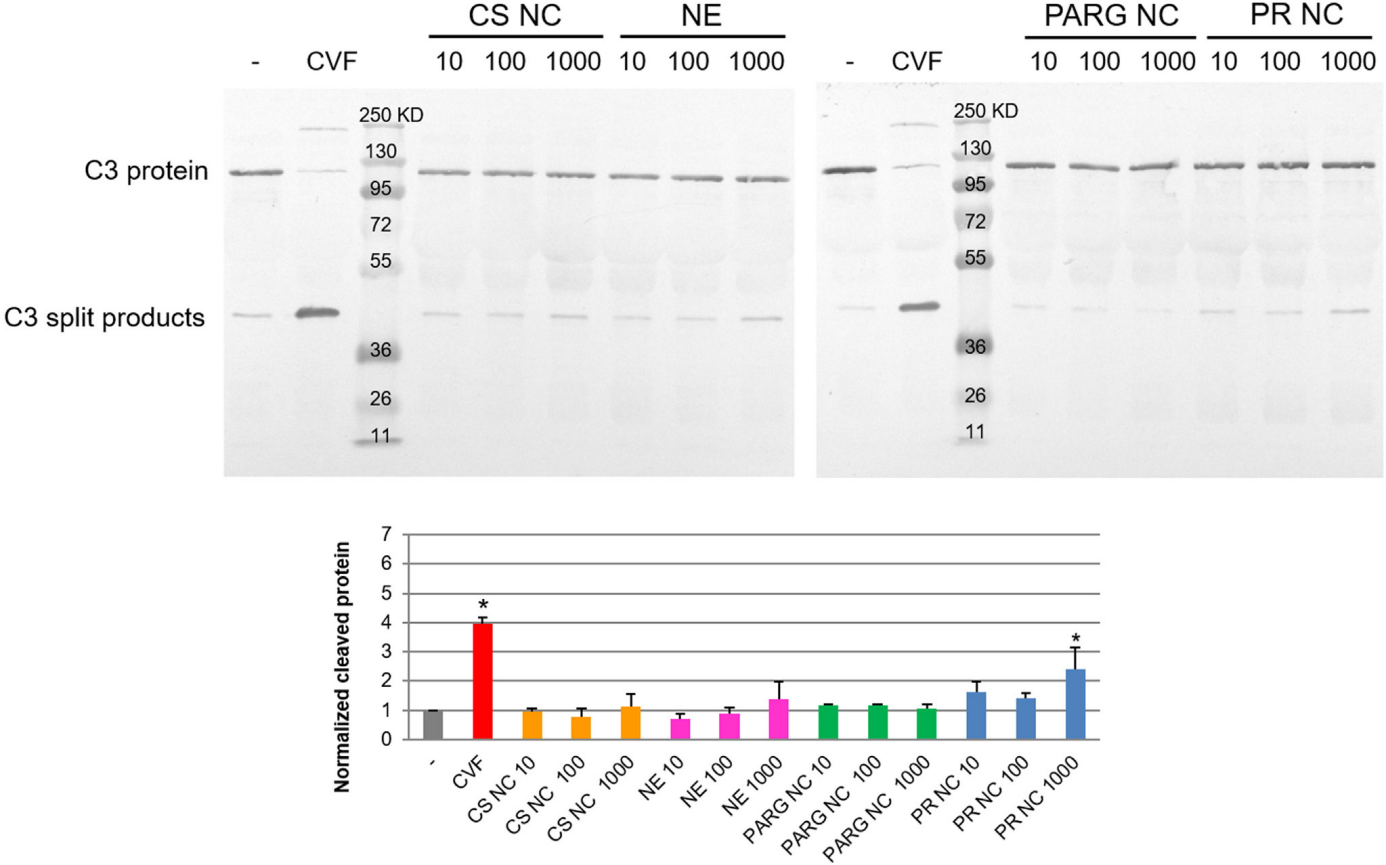
Complement activation of human plasma induced by the nanostructures. Different concentrations of the prototypes were tested by Western blot: 10, 100, and 1,000 µg/mL. Phosphate-buffered saline and cobra venom factor (CVF) were selected as negative and positive controls. Abbreviations: NE, nanoemulsion; CS NC, chitosan nanocapsule; PARG NC, polyarginine nanocapsule; PR NC: protamine nanocapsule. Bands corresponding to the C3 protein and C3 split products are indicated. Molecular weight controls are expressed in kilodalton. The lower graph corresponds to the intensity of the lower band (C3 cleaved) normalized to the negative control. The median ± SD is represented (*n* = 3). *Significant differences between negative and positive (CVF) controls or nanocapsule treatments.

Analysis of the C3 degradation by Western blot showed that PR NCs slightly induced the activation of the complement cascade. The highest rate of activation was achieved at the highest concentration tested (1 mg/mL of theoretical concentration of protamine), and the degradation rate was double that found in the negative control (Figure 1). This result was confirmed by ELISA using the quantification of the C5a factor levels, which were two times higher in the plasma exposed to the NCs than in the untreated negative control (data not shown).

It has been previously reported that the nanoparticle surface composition plays a crucial role in the complement cascade activation: the presence of a high surface density of amino groups in the polymer chain can enhance the interaction with the C3b α-chain (23). PR and PARG are guanidine-rich polymers, and they would be expected to activate the complement cascade. By contrast, the results on the complement activation properties of chitosan reported to date are quite variable (24, 25), probably due to the different types and sources of chitosan on the market. In our case, activation was not observed after contact between this polymer and the complement cascade proteins.

Western blot analysis showed that only PR NCs, and not PARG NCs, were able to achieve significant levels of the C3 split products at the highest concentration tested. This finding suggests that another mechanism, besides the interaction with the C3b α-chain, may be contributing to the activation of the complement cascade. This difference could also be attributed to a higher percentage of adsorption of the PR when compared with the PARG. Further investigation need to be perform to validate this hypothesis. Although little is known about the mechanism of complement activation induced by arginine-rich polymers, it has previously been reported that PR, which is commonly used to neutralize the effect of heparin after cardiopulmonar bypass, can induce complement cascade activation through the classic route when heparin–PR complexes are formed (26).

### *In Vitro* Cell Studies

#### Cell Viability

**Figure 2:**
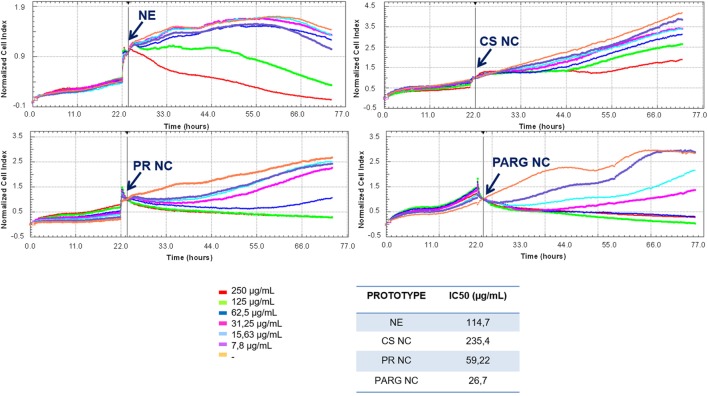
Effect of the prototypes on the viability of Raw 264.7 cells. Cells were cultured until their exponential phase, and then the nanostructures were added (indicated with an arrow) at different concentrations. IC50 was calculated with RTCA Software 1.2.1. Abbreviations: NE, nanoemulsion; CS NC, chitosan nanocapsule; PR NC, protamine nanocapsule; PARG NC, polyarginine nanocapsule.

Six different concentrations of the nanosystems (from 250 to 7.8 µg/mL) were used to evaluate the cell viability. As shown in Figure 2, a dose-dependent trend was observed for all formulations. PARG NCs reduced cell viability to a greater extent than the other prototypes, with an IC50 close to 30 µg/mL, followed by PR NCs > NE > CS NCs. A major reduction in the cell viability has been previously reported with NCs presenting PARG in the surface composition, being this toxicity reported as dose dependent and influenced by the composition of the nucleus (27). In addition, it has previously been reported that the toxic effect induced by nanoparticles decreases with increasing particle size (28). This may explain why CS NCs are the prototype that reduces less the cell viability. In the case of the NE, this prototype was only able to reduce the cell viability at the higher concentrations tested: the negative charge on the surface may play an important role in the reduction of cell viability (29, 30).

#### Cellular Uptake and ROS Production by Macrophages

**Figure 3:**
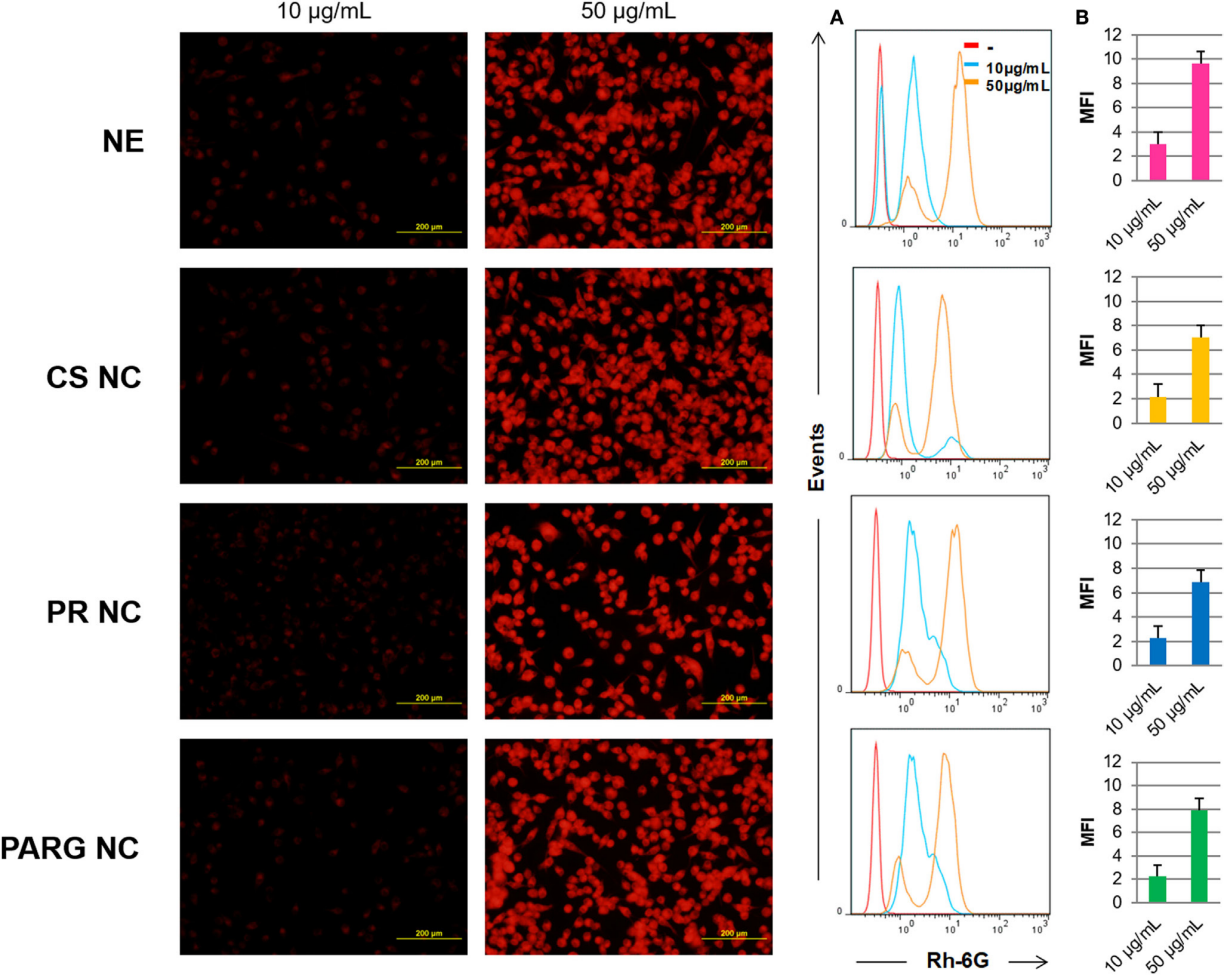
Internalization of the fluorescent nanostructures by Raw 264.7 cells. Left: cells incubated with the fluorescent prototypes (images taken on a fluorescence microscope) at 10 and 50 µg/mL. Right: cells analyzed by flow cytometry **(A)** by histograms **(B)** by the median of the fluorescence intensity (MFI). Abbreviations: NE, nanoemulsion; CS NC, chitosan nanocapsule; PR NC, protamine nanocapsule; PARG NC, polyarginine nanocapsule.

**Figure 4:**
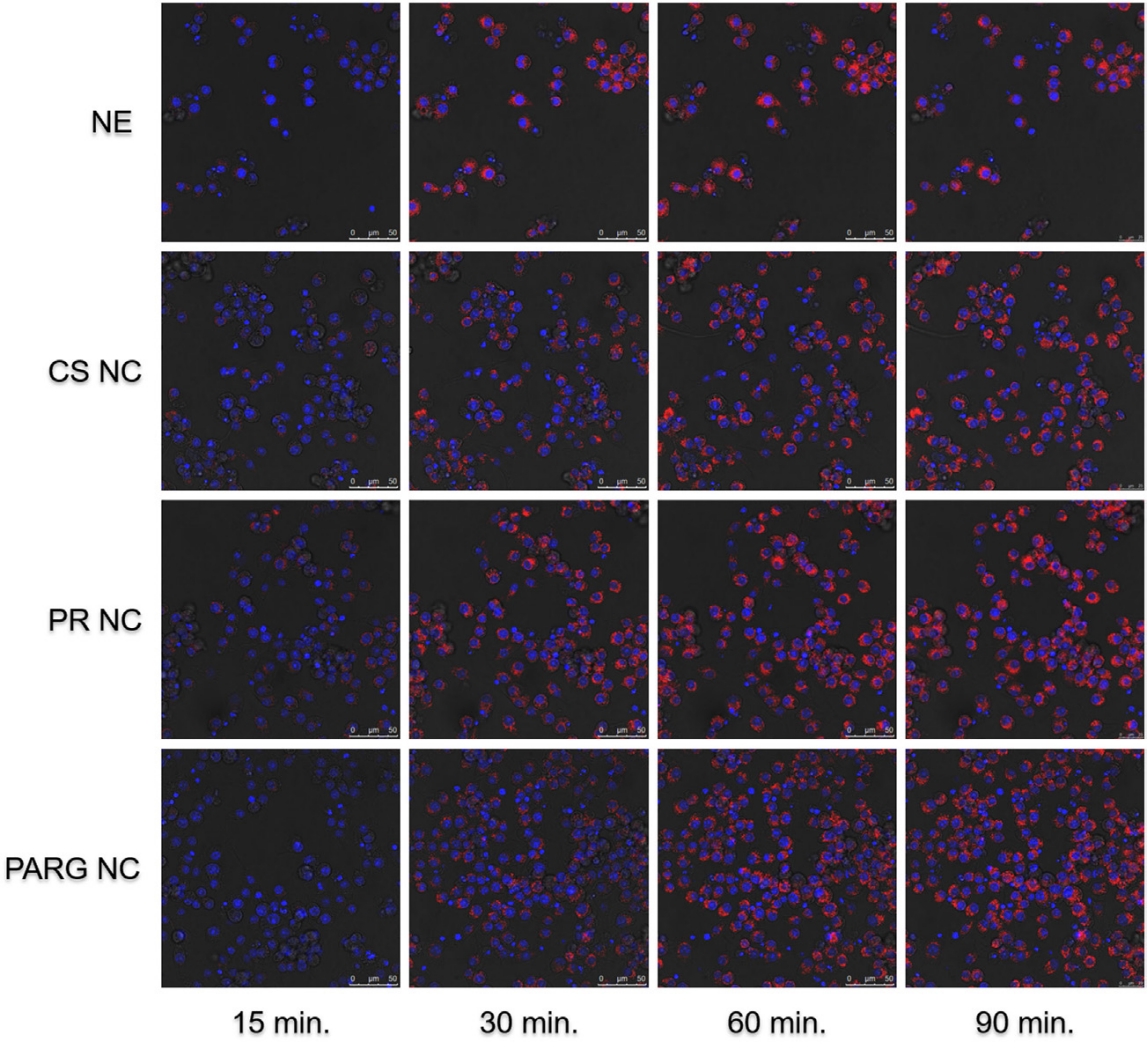
Kinetic internalization study of the labeled nanostructures performed on a confocal microscope [red channel: rhodamine 6G (labeled nanocapsules); blue channel: DAPI (nucleus)]. Raw 264.7 cells were incubated in the presence of the nanosystems, and images were acquired every 5 min. Images at 15, 30, 60, and 90 min are shown. Abbreviations: NE, nanoemulsion nanocapsule; CS NC, chitosan nanocapsule; PR NC, protamine nanocapsule; PARG NC, polyarginine nanocapsule.

Fluorescence microscopy and flow cytometry results (Figure 3) indicate that all prototypes were efficiently internalized by the cells in a dose-dependent manner. A real-time uptake study using Confocal Laser Scanning Microscopy and the High Content Screening Automation HCS A module was performed by incubating macrophages with fluorescent NCs during 90 min. All of the prototypes were quickly internalized, and the fluorescence was detected after 15 min from the starting time point of the experiment (Figure 4). The fluorescence intensity increased up to the last time point of the experiment.

It has been reported in the literature that particulate delivery systems are commonly taken up by phagocytic cells and that this internalization is dependent on particle size, composition, and surface charge (31–33). In general, charged large particles are internalized more rapidly than small neutral particles, with the cationic prototypes being particularly prone to uptake within charged systems (34). In this study, all the formulations are charged, positively or negatively, and have proven to be efficiently internalized. The charge, regardless of its sign, may have facilitated capture by macrophages: while cationic nanoparticles would have easily interacted with anionic cell surfaces, the internalization of anionic NEs may have occurred through the non-specific binding of the particles on cationic domains followed by endocytosis (35). It has been described in other polymeric nanomaterials, that once they are internalized in endosomes, they go to lysosome where they are processed. They can induce ROS production, and eventually, trigger the immune response (36–38). In addition, it has been reported that ROS production by the dendritic cells plays an important role in the antigen presentation in that it stimulates the cross-presentation in these cells and therefore it enhances the CD8^+^ T lymphocyte responses (39). As a consequence, ROS production in the promyelocytic cell line HL60 was studied as a measure of cell activation after the internalization of these prototypes, where the cell activation was at the same time an indicator of the initiation of the adaptive immune response.

The ROS production was studied after cell exposure to nanostructures during 1 or 12 h (Figure S3 in Supplementary Material). All the prototypes induced the production of ROS in a dose- and time-dependent manner. It has been previously reported that the induction of ROS by inorganic nanoparticles could be a mechanism for the promotion of cell death (40). However, little is known about ROS production by polymeric nanoparticles as a mechanism of cell defense against infections.

#### Cytokine Profile Evaluation

Cytokines are peptides, small proteins, or glycoproteins that play an important regulatory role in several biological processes. They are produced mainly by immune cells, but also by several other cell types. Cytokines are the main messengers between cells of the immune system and they regulate the innate and adaptive responses and also modulate the inflammatory response (41). In this study, our main goal was to investigate if the NCs, to be used as antigen carriers, are inherently able to stimulate cytokine secretion by hPBMCs.

It can be seen from the results in Table 2 that the PR and PARG NCs have a slightly greater tendency to induce the production of pro-inflammatory cytokines than CS NCs or the control NE. The main difference was observed in the secretion of TNFα, where two out of the three donors responded in the case of PR and PARG NCs, only one out of three donors was positive for CS NCs, and no positive response was observed for the control NEs. Although these responses are not remarkable, they are in agreement with previous studies in which it was found that protamine and PARG are able to induce the production of TNFα; PR was included in a nanoparticulate form and incorporated CpG (42), while in the case of PARG it was the bulk material (43). On the other hand, it has also been reported that cationic particles are more efficient at inducing inflammatory responses than anionic or neutral particles (34). This observation is consistent with the overall cytokine profiles observed for the three types of NCs. By contrast, the anionic NE was only able to induce the production of IL-8, a cytokine that is basally produced at high levels (Table 2). The differences in the cytokine secretion profiles for the three cationic prototypes could be explained by the smaller size of PR and PARG NCs when compared with CS NCs: the higher specific surface area may contribute to a more efficient interaction with the mononuclear cells (44).

**Table 2:** Cytokine production by human peripheral blood mononuclear cells incubated with nanocapsules at two doses (10 and 100 µg/mL).

		μg/mL	NE	CS NCs	PR NCs	PARG	LPS-PHA
Th1 profile	IL-2	10	–	–	–	–	++
		100	–	–	–	++1/3	
	IL-12p70	10	–	–	–	–	+++
		100	–	–	–	–	
	IFN γ	10	–	–	–	++1/3	+++
		100	–	–	–	–	
	TNF β	10	–	–	–	–	+
		100	–	–	–	–	
	TNF α	10	–	–	–	–	+++
		100	–	+1/3	+2/3	+2/3	
Th2 profile	IL 4	10	–	–	–	–	+
		100	–	–	–	–	
	IL 5	10	–	–	–	–	+
		100	–	–	++1/3	++1/3	
	IL 10	10	–	–	–	–	++
		100	–	–	+1/3	+1/3	
Other pro-inflammatory cytokines	IL 1β	10	–	–	–	–	+
		100	–	+1/3	+	+	
	IL 6	10	–	–	–	–	+++
		100	+1/3	++	+++	++	
	IL 8	10	+1/3	–	+1/3	+2/3	+
		100	+	+	+	+	

*NE, nanoemulsion; CS NC, chitosan nanocapsule; PR NC, protamine nanocapsule; PARG NC, polyarginine nanocapsule*.

*The positive control was lipopolysaccharide (LPS) (1 µg/mL) with phytohemagglutinin (PHA) (10 µg/mL)*.

*+: 1–10; ++: 10–100; +++: 100–1,000; ++++: >1,000-fold higher than negative control (cells incubated in culture media)*.

*+N/tested: number of positive responsive persons/three donors tested*.

The cytokine profile induced by PR and PARG prototypes is dominated by IL-6, Il-1β, IL-8, and TNFα (at the highest dose tested). All of these cytokines are mainly produced by macrophages when they are exposed to inflammatory stimuli (45). While IL-8 is a potent chemotactic factor for lymphocytes and neutrophils that is often released during phagocytosis, IL-6 induces B cell differentiation and increases antibody titers (46, 47). In addition, IL-1β is released upon activation of macrophages *via* Caspase 1 pathway, and similarly to this one, TNFα is also an endogenous pyrogen that is produced and released at the early stages of the immune response to infections (45). Both prototypes have proved to be efficiently internalized by phagocytic cells (Figures 3 and 4), hence a high internalization rate during the first steps of the immune response may have contributed to the activation of human monocytes, leading to the release of all these pro-inflammatory cytokines generating an adequate scenario to initiate a specific immune response.

#### Activation Markers

Human CD71 (transferrin receptor) and class II molecules of the major histocompatibility complex (HLA-DR) are membrane markers that can be modulated during cell activation. Transferrin receptor is expressed at different levels in many cell types and increases its expression in activated cells such as T and B lymphocytes, because this mechanism is used to import extracellular iron for metabolic needs (48, 49). HLA-DR is constitutively expressed in B cells and it has been shown to be down regulated under certain stimuli. The modulation of this marker changes depending on the stage of differentiation of the B cells upon its activation. While an increase during the first contact with stimuli is commonly observed, a decrease occurs during the last steps of differentiation (50–52). On the other hand, T cells do not express HLA-DR unless they are activated (53).

**Figure 5:**
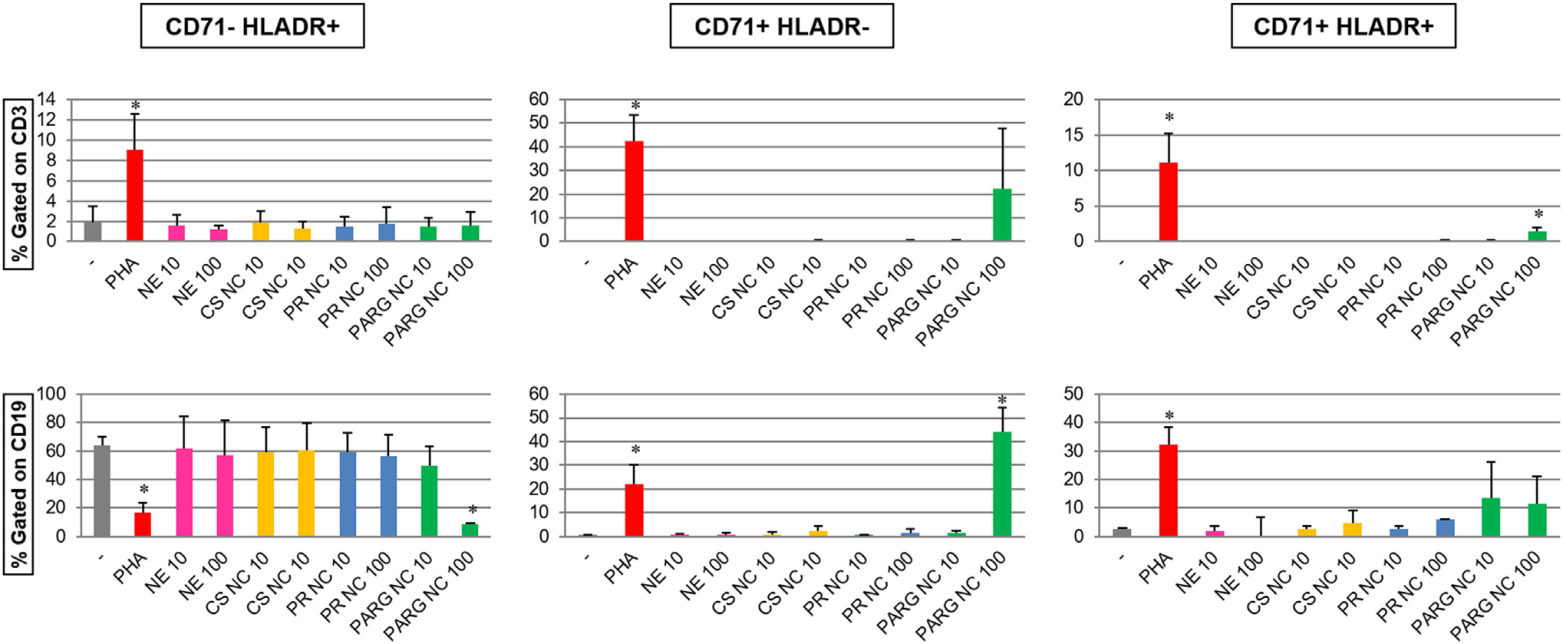
Modulation of activation markers (CD71 and HLA-DR) in gated T (CD3^+^) and B (CD19^+^) lymphocytes (top and bottom graphs, respectively) from human peripheral blood mononuclear cells (hPBMCs) incubated with the prototypes (at 10 and 100 µg/mL). Percentages of CD71^–^HLADR^+^, CD71^+^HLADR^–^, and CD71^+^HLADR^+^ cells are represented. The results are the average of three independent experiments with hPBMCs from three different healthy donors (*p < 0.05).

The expression of the activation markers CD71 (transferrin receptor) and HLA-DR in T (as CD3^+^) and B (as CD19^+^) cells incubated with the different nanostructures at two different doses (10 and 100 µg/mL) was evaluated by flow cytometry (Figure 5). The results indicate that only PARG NCs were able to increase the percentage of CD71^+^HLA-DR^+^ T cells and also the expression of CD71 in B cells. Furthermore, PARG NCs induced the modulation of HLA-DR in B cells. While this effect seems to correlate to cell activation, it cannot be excluded that a cytotoxic effect could have been the responsible for this modulation in the expression of these markers as consequence of exposure to damage-associated molecular patterns (DAMPs, danger signals, or alarmins). In fact, these molecules have been described as the possible responsibles for the adjuvant effect of other molecules such as alum (54).

These results are in agreement with the cytokine data, where PARG NCs induced the production of different cytokines in hPBMCs. A synergistic effect of all of these cytokines may play a crucial role in the lymphocyte activation and subsequently in the modulation of markers such as CD71 or HLA-DR.

#### Routes of Activation. MAP Kinases

Mitogen-activated protein kinases (MAPKs) are a protein family that is involved in several processes such as gene expression, mitosis, cell migration, metabolism, and programmed cell death. MAPKs are activated through the phosphorylation of certain residues and, in turn, they activate their target proteins, including other protein kinases, transcription factors, or cytoskeleton proteins.

The MAPK families that are most widely reported in the literature are the ERK, the c-Jun NH2-terminal kinases (JNK), and the p38 family. While ERKs are involved in the regulation of processes such as mitosis or meiosis, JNK are mainly involved in the control of programmed cell death or apoptosis. On the other hand, p38 participates in the immune response activation (55). In addition, the NFκB is a transcription factor that plays an important role in the regulation of certain immune responses by modulating the expression of receptors or surface adhesion molecules, inflammatory cytokines, and chemokines. Furthermore, it is involved in the response to stress signals and in cell survival (56, 57).

In this study, we investigated the capacity of PR and PARG NCs to induce the secretion of MAPK and NFκB in two different cell lines, namely, the Jurkat (human leukemic T-cell line) and the Hmy (human B Lymphoblast cell line) cell lines. It has previously been reported that activation of the NFκB pathway leads to the degradation of the IKBα inhibitor and therefore a decrease in IKBα was expected in these cells.

**Figure 6:**
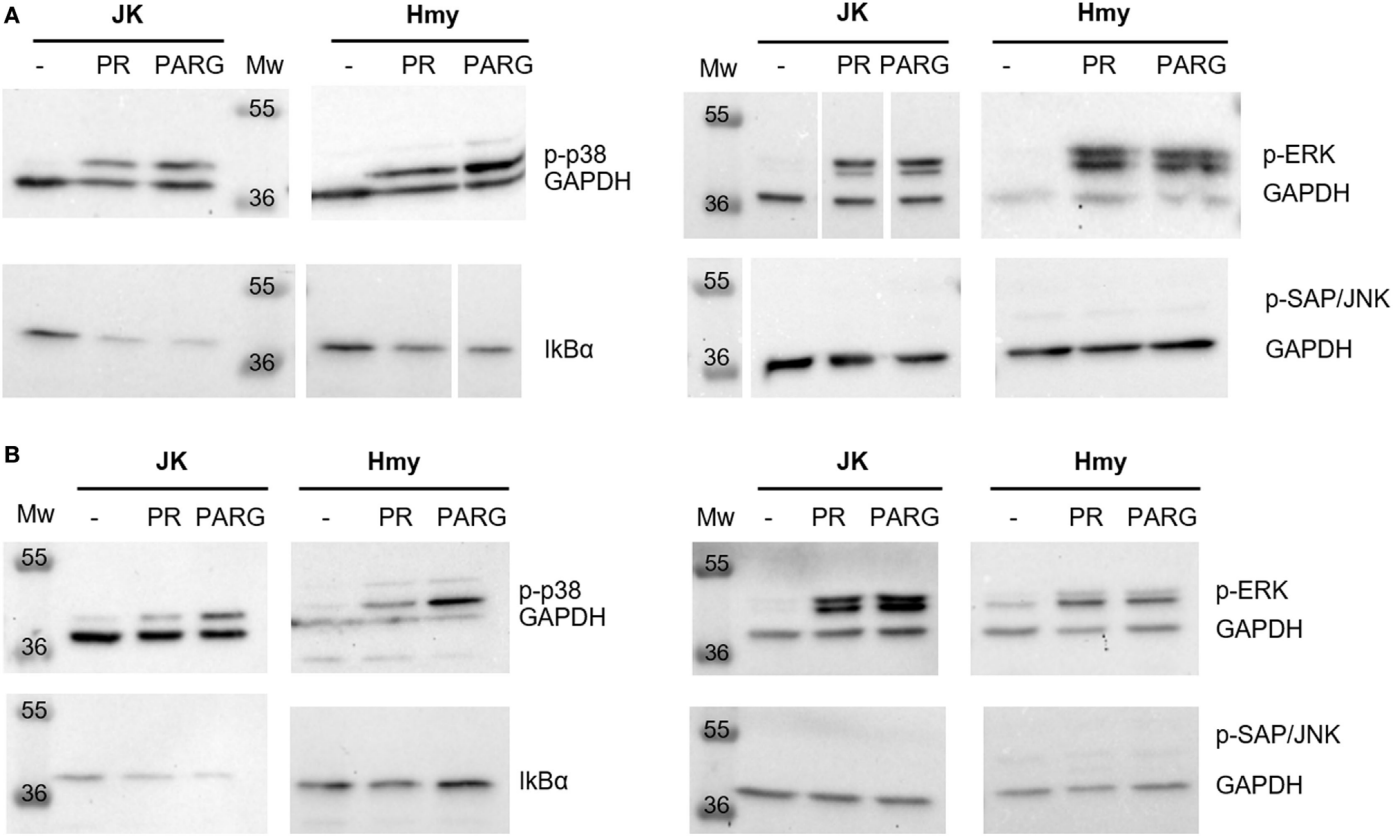
Reduction in IκBα expression and activation of mitogen-activated protein kinases [p-extracellular signal-regulated kinase (ERK), p38, and p-SAP/c-Jun NH2-terminal kinases (JNK)] and the nuclear factor κB pathway in Jurkat and Hmy cell lines induced by PR NCs and PARG NCs after 1 h **(A)** or 3 h **(B)** of incubation. GAPDH was used as a loading control. White space between lanes shows the cut point in membranes.

The Western blot analysis only showed a slight increase in the phosphorylation levels of the p38 family after 3 h in contact with PARG NCs (Figure 6). Considering Hmy and Jurkat cell lines as representative B and T cells, respectively, of the main PBMCs, this result could correlate with the observed capacity of these NCs to induce the production of Il-1β, IL-6 and IL-8 in human PBMC, as it is known that the p38 family is activated in response to different inflammatory cytokines and the triggering of different immune responses (55, 58, 59). In addition, p-p38 has also been shown to be involved in the proliferation of Th1 cells (60). This explains why p38 is activated mainly by PARG NCs, as this NCs formulation showed a Th1 profile characterized by the production of TNFα.

In the case of ERK, both prototypes, PR and PARG NCs, induced its activation in Hmy and Jurkat cells, with this effect being time dependent only in Jurkat cells (Figure 6). ERK is activated in response to several cytokines, and it is involved not only in cell survival but also in cell differentiation (55). In addition, ERK phosphorylation is an important step during both T and B cell activation (61, 62) and in the Th2 differentiation process (63).

Regarding p-SAP/JNK, activation was not detected upon contact with any of these prototypes (Figure 6). This fact can be explained by the pro-apoptotic activity of the p-SAP/JNK family: the lack of activation was expected (58, 64) since none of the prototypes showed a cytotoxic effect at low doses and in short periods of time.

The activation of NFκB was detected by the decrease in its IkBα inhibitor levels (Figure 6). This fact was observed in both Jurkat and Hmy cells and with both protamine NCs and PARG NCs. NFκB is activated by a wide range of signals such as antigen receptors, pattern-recognition receptors, and different TNF and IL-1 receptors. This activation regulates not only the innate but also the adaptive immunity and different inflammatory responses (65). The decrease in the IkBα levels in both Jurkat and Hmy cell lines upon contact with this immune stimulator was expected considering that this transcription factor is essential to trigger some processes related to immune cell activation.

In light of the results obtained, it can be concluded that PR and PARG NCs are able to induce the phosphorylation of p38 and ERK, as well as the degradation of IκBα in both T and B cell lines, with PARG NCs having a slightly superior activation capacity.

Overall, data obtained by *in vitro* studies, postulate PR and PARG NCs as the most promising prototypes as antigen delivery systems. On the other hand, NE and CS NC have not demonstrated to be strong inducers of immune responses and do not activate the complement cascade, thus they could be useful as carriers intended to avoid the immune system recognition, for example to target cells for gene therapy (66).

### *In Vivo* Studies

Taking into account the results obtained in the *in vitro* studies, PARG and PR NCs were selected for the *in vivo* evaluation of their capacity to induce the production of an immune response against a model antigen, rHBsAg. To this end, groups of five Balb/C female mice were intramuscularly immunized with two doses of rHBsAg adsorbed onto PR NCs and PARG NCs or alum. The humoral-specific immune response (anti-rHBsAg serum IgG) and the type of immune response elicited (measured by the IgG1/IgG2a ratio) were both analyzed. In addition, a panel of several genes involved in immune responses was studied *ex vivo* in cells from immunized mice.

#### Immune Response Elicited by Immunostimulating rHBsAg-Loaded NCs

**Figure 7:**
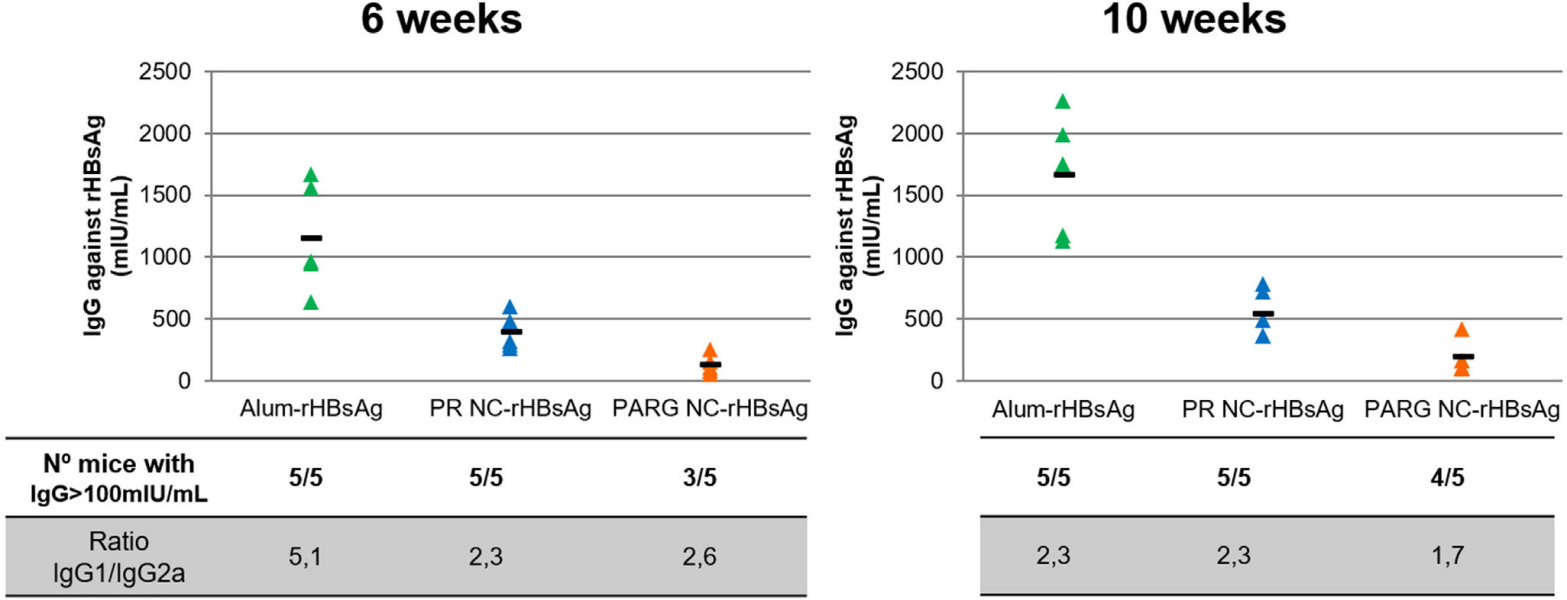
Systemic immune response upon intramuscular immunization with rHBsAg PR NCs and PARG NCs. The top graphs represent individual IgG concentrations of anti-HBsAg (expressed in mUI/mL) in each mouse (triangles) and the average of five mice (dash) at 6 weeks (left graph) and 10 weeks (right graph) after the first administration. The number of mice with antibody levels over 100 mIU/mL and the IgG1/IgG2a ratios at different time points are also represented in the lower table.

It can be seen from the results in Figure 7 that PR NCs and PARG NCs were able to elicit antigen-specific antibody levels at 6 weeks after immunization, with levels close to 400 and 130 mIU/mL, respectively. These levels increased slightly to 500 and 200 mIU/mL, respectively, after 10 weeks. It is worthy to note that in both cases, seroprotection levels (>100 mUI/mL) were achieved, although this increase was not as high as the one observed in control mice immunized with alum-rHBsAg (from 1,100 to 1,600 mUI/mL) (Figure 7).

Despite the response induced by the marketed alum-rHBsAg vaccine was much higher, the average levels achieved by PR NCs and PARG NCs were over 100 mIU/mL, which has been established as the threshold of antibody protective levels in humans (67). It can also be stated that PR NCs showed better immunostimulant properties than PARG NCs as they induced higher specific antibody levels in mice (5/5). This difference can be explained by the higher ability of PR NCs to activate the complement cascade compared with PARG NCs, which may play a crucial role in the adjuvant effect of the NC. Thus, independently on the NCs under study, the immune response induced by both prototypes could be due to a combination of several routes such as p38 and ERK. Moreover, the lack of a strong activation of the NFκB pathway could be related to the moderate *in vivo* immune response obtained in the presence of NCs.

#### Modulation of the Immune Response

To determine the type of response induced by these prototypes, the IgG1/IgG2a ratio was calculated for both prototypes. These two IgGs were selected because while the IgG2a subtype is related to cellular immune responses mediated by Th1 lymphocytes, IgG1 is mostly produced after Th2 activation, which mediates the humoral immune response.

Both humoral and cellular responses were elicited by both prototypes. The highest levels detected for Th2, with ratios ranging from 2.6 to 1.7. While this ratio did not change over time for PR NCs, it decreased slightly for PARG NCs, which indicates that an increase in Th1 response had occurred (Figure 7). This finding is in agreement with the combination of cytokines produced by hPBMCs upon contact with PARG NCs and also due to the activation of both p-ERK and p-p38, which are commonly involved in Th2 and Th1 responses, respectively (60, 63). The same trend was observed in the control group, although in this case the initial IgG1/IgG2a ratio was higher (close to 5). This is in agreement with the known fact that alum is a very effective Th2 activator (68).

#### Gene Expression Assays in Restimulated Splenocytes From Immunized Mice

To understand the mechanism involved in the immunostimulant properties of PARG NCs and PR NCs, the expression of different genes was studied by qPCR. For this purpose, splenocytes from immunized mice were restimulated with the HBsAg during 12 or 24 h. RNA from these cells was extracted to analyze the expression of genes involved in apoptosis and inflammation (Caspase 1, 3, 8, and 9; Fas and Bcl2) of different cytokines (IL-10, 1L-4, IL-2, INF-γ, IL-6, TNF-α, IL-1β, and IL-17) and of certain activation markers (CD74, B2M, CD71, CD69, CD154, CD80, and CD86). The results of these studies are summarized in Table 3, and the values highlighted in red show an increase in the RQ value greater than 2. Significant differences were only registered for IL17A in mice immunized with PR NCs-HBsAg both 12 and 24 h after restimulation. A minor increase was also observed in IL-1β levels in the same group after 24 h of restimulation.

**Table 3:** Changes in the expression of selected genes in splenocytes from mice immunized with alum-rHBsAg, PRNC-rHBsAg, and PARGNC-rHBsAg restimulated for 12 or 24 h with rHBsAg.

Gene	Name	RQ (12 h)			RQ (24 h)		
		Alum-rHBsAg	PRNC-rHBsAg	PARGNC-rHBsAg	Alum-rHBsAg	PRNC-rHBsAg	PARGNC-rHBsAg
B2M	Beta-2-microglobulin	0.93	0.71	0.91	0.98	1.28	0.82
TNF	Tumor necrosis factor	1.12	0.95	1.16	1.16	1.38	0.74
IL10	Interleukin 10	0.80	0.69	0.89	0.76	1.17	0.65
IL4	Interleukin 4	1.10	0.73	0.88	1.07	1.62	0.79
IL2	Interleukin 2	1.28	0.92	0.96	0.92	1.58	0.73
INFγ	Interferon gamma	0.92	1.03	1.19	0.84	1.26	0.69
IL6	Interleukin 6	0.93	0.81	1.04	0.89	1.24	0.72
IL1β	Interleukin 1 beta	1.68	1.34	1.40	1.20	2.06	1.13
IL17A	Interleukin 17A	1.72	2.30	0.91	2.18	3.11	0.46
CASP1	Caspase 1	0.94	0.81	1.01	1.43	1.36	1.05
CASP3	Caspase 3	0.88	0.80	0.91	1.33	1.26	0.88
CASP8	Caspase 8	1.02	0.80	0.92	1.18	1.14	0.89
CASP9	Caspase 9	0.82	0.77	0.83	1.43	1.25	0.91
Bcl 2	B-cell lymphoma 2	0.73	0.68	0.83	1.11	1.00	0.81
FADD	Fas-associated protein with death domain	0.91	0.76	0.74	1.74	1.21	0.89
CD154	Cluster of differentiation 154	0.63	0.64	0.70	0.92	0.94	0.72
CD69	Cluster of differentiation 69	1.12	0.91	0.99	1.24	0.90	0.65
CD74	Cluster of differentiation 74	0.91	0.96	1.09	1.48	1.35	1.06
TFRC	Transferrin receptor	0.88	0.80	0.90	0.96	1.32	0.90
CD86	Cluster of differentiation 86	1.09	0.83	0.94	0.02	1.50	1.00
CD80	Cluster of differentiation 80	0.69	0.91	0.88	1.19	1.30	0.79

*Values in this table represent the RQ with respect to the control (splenocytes from mice immunized with phosphate-buffered saline 1× and restimulated *in vitro* with rHBsAg) calculated from independent data for 3 mice per group*.

*RQ values greater than 2 are represented in red (% CV of ΔCt < 25)*.

*RQ, relative quantification; PR NC, protamine nanocapsule; PARG NC, polyarginine nanocapsule; rHBsAg, recombinant hepatitis B surface antigen*.

IL-17A is a cytokine produced by Th17 cells and by other immune cells, including γδ T cells, NKT cells, NK cells, neutrophils, and eosinophils. In fact, one of its functions is to connect innate and adaptive immune responses. IL-17A is a pro-inflammatory cytokine that induces the production of other cytokines and chemokines, which are crucial in the recruitment, activation, and migration of neutrophils to the site of inflammation. It has been reported that this cytokine is also involved in the development of germinal centers (GC) (69). IL-1β, which is produced through the NALP3 inflammasome route and whose release can be initiated by the complement cascade activation (70), is one of the major mediators of inflammation and it is the main known endogenous pyrogen. IL-1β is also very important in the initiation of innate immune responses and, as a consequence, it has a critical role in the control of pathogenic infections (71). Both cytokines IL-17 and IL-1 have been proposed as the main mediators of the adjuvant effect of several vaccines (72–74). The induction of IL-17 and IL-1β production by PR NCs in cells from immunized mice may have an important role in initiating the immune response after vaccination. These could induce an inflammatory response that would create the appropriate environment for the development of a specific immune response.

PR NCs have proved to be efficient antigen carriers *in vivo* using the HBsAg as a model antigen. We postulate that the incorporation of immunostimulant molecules into this formulation, such as imiquimod, CpG, polyI:C (13, 14, 75), could lead to the design of a prototype able to induce an enhanced immune response, even stronger than that induced by alum, and also to an effective system that could modulate this immune response toward a Th1 profile.

## Conclusion

The results underline the potential of arginine-rich NCs as antigen delivery carriers. The systematic analysis performed in this study provides valuable information regarding the feasibility of modulating the immunostimulatory properties of NCs by selecting the nature of the polymer shell. This information could help in the design and development of novel nanocarriers for vaccine delivery, taking as a starting point the physicochemical properties of PR NCs, which was the prototype that showed the best immunological profile in the *in vivo* evaluation.

## Ethics Statement

Institutional ethics approval to work with human samples from healthy donors was obtained from the Ethics Committee for Clinical Research (Xunta de Galicia, Spain, 2013/272). All participants included in the study gave their written informed consent. All protocols developed with mice were adapted from the guidelines of the Spanish regulations (Royal Decree 53/2013) regarding the use of animals in scientific research and under the approval of the Ethical Committee of the University of Vigo.

## Author Contributions

MP and EP have contributed to the design, acquisition, analysis, and interpretation of data; the drafting and the revision of the work. JG-A, BS-C, and RS-V have contributed to the design, acquisition, analysis, and interpretation of data for the work. NC, MA, and ÁG-F have contributed to the design, the drafting, and the revision of the work.

## Conflict of Interest Statement

The authors declare that the research was conducted in the absence of any commercial or financial relationships that could be construed as a potential conflict of interest.
