# Supplementary material for: Polymeric Nanocapsules for Vaccine Delivery: Influence of the Polymeric Shell on the Interaction With the Immune System
Source: Front Immunol. 2018 Apr 19;9:791. doi: 10.3389/fimmu.2018.00791 (PMC5916973; doi:10.3389/fimmu.2018.00791)
Supplement: Supplementary file 1 [file data_sheet_1.docx]

Supplementary Material

Polymeric nanocapsules for vaccine delivery: influence of the polymeric shell on the interaction with the immune system

Mercedes Peleteiro#, Elena Presas#, Jose Vicente González-Aramundiz, Beatriz Sánchez Correa, Rosana Simón-Vázquez, Noemi Csaba, M.J. Alonso*, África González-Fernández*

^#^These authors contributed equally to this work.

* Correspondence:

África González Fernández: africa@uvigo.es

M. J. Alonso: Mariaj.alonso@usc.es

# Supplementary Material

- 1. Evaluation of the loading efficiency and release profile of a fluorescent dye

In order to label the nanocarriers, preliminary studies were conducted with three different chromophores (DiD, rhodamine B and rhodamine 6G). The studies were performed with the NE, CS NC and PR NC, assuming that both the encapsulation efficiency and the release profile of the fluorescent dyes were the same for PR NC and PARG NC due to their similar arginine-rich structure. All prototypes showed high encapsulation efficiency for rhodamine B and DiD (close to 90%) but a quick and complete release was observed for both chromophores (data not shown). In contrast, the encapsulation efficiency of rhodamine 6G was estimated to be 30%, but leakage was not observed upon storage for 3 days. Moreover, burst release was not observed during the *in vitro* assays (2 h, 37 °C in PBS). As a result, rhodamine 6G was selected for further studies (**Supplementary** **Figure 1**).

**Supplementary Figure 1:** Release (expressed in percentage) of rhodamine 6G (50 µg/mL) from the different labelled nanosystems incubated in PBS for 0.5, 1 and 2 hours. NE: nanoemulsion NC: nanocapsules; PR: protamine; CS: chitosan.

Regarding the physico-chemical properties of the nanostructures after the incorporation of 50 µg/mL of rhodamine 6G, both the NE and the PR NCs were able to maintain their original size, zeta potential and low polydispersity index upon integration of the molecule into the nanoparticle structure (**Supplementary Table 1**). In the case of CS NCs, a substantial increase in size was observed after the incorporation of the dye. This effect can be explained by the inability of the nucleus to accommodate this polymeric shell after dye incorporation, thus leading to an increase in size of the prototype.

**Supplementary Table 1:** Physico-chemical characterization and encapsulation efficiency of the nanoemulsion, protamine and chitosan nanocapsules loaded with rhodamine 6G (50 µg/mL).

| Formulation | Size  (nm) | Polydispersity | Zeta potential (mV) | Encapsulation (%) |
| --- | --- | --- | --- | --- |
| NE | 362 ± 39 | 0.2 | –10 ± 1 | 36 ± 4 |
| PR NCs | 342 ± 27 | 0.2 | +27 ± 4 | 23 ± 8 |
| CS NCs | 648 ± 29 | 0.3 | +59 ± 3 | 22 ± 5 |

Abbreviations: NE: nanoemulsion NC: nanocapsule; PR: protamine; CS: chitosan

- 1. **Freeze-drying of the nanocapsules**

Upon preparation, nanocapsules can undergo different processes (aggregation, dissociation, polymer degradation etc.) that could destabilize their structure. Their conversion into a dry powder is one of the most common strategies to enhance their physico-chemical stability. In addition, freeze-drying represents an attractive approach to avoid the need to maintain the cold-chain for vaccine transport and storage (1). However, this procedure could lead to partial destruction of the polymeric coating of the nanocapsules due to the physical stress suffered during the freezing step, or due to the formation of ice crystals that can lead to aggregation process. In order to obtain a stable dry powder product, a range of concentrations of the nanosystems and different protective excipients were evaluated. As shown in **Supplementary Figure 2**, the results indicate that the particle size and zeta potential of the nanocapsules were maintained after the resuspension of the freeze-dried products of the different nanosystems (0.75% w/v) prior incorporation of sucrose as cryoprotectant (10% w/v) (**Supplementary Figure 2**).

**Supplementary Figure 2:** Size variations of the different prototypes after the lyophilization processes at a nanoparticle concentration of (0.75% w/v) and sucrose as cryoprotectant (10% w/v).

1. Chen GaW, W. Role of freeze drying in nanotechnology. Drying Technology.25(1-3):6.

- 1. **ROS production by macrophages**

Supplementary Figure 3. ROS production by HL60 cells after 1 or 12 hours of incubation with nanocapsules. The median of the fluorescence intensity normalized to the negative control (unstimulated cells) is represented. The average ±SD is represented (n = 3). * Significant differences between negative control (–) and positive control (PMA) or upon contact with the prototypes (* p<0.05).
